# The Role of Innate Immune Cells in the Prediction of Early Renal Allograft Injury Following Kidney Transplantation

**DOI:** 10.3390/jcm11206148

**Published:** 2022-10-18

**Authors:** Nora Jahn, Ulrich Sack, Sebastian Stehr, Maria Theresa Vöelker, Sven Laudi, Daniel Seehofer, Selim Atay, Panagiota Zgoura, Richard Viebahn, Andreas Boldt, Hans-Michael Hau

**Affiliations:** 1 Department of Anesthesiology and Intensive Care Medicine, University Hospital of Leipzig, 04103 Leipzig, Germany; 2 Institute of Clinical Immunology, Medical Faculty, University of Leipzig, 04103 Leipzig, Germany; 3 Department of Visceral, Transplantation, Vascular and Thoracic Surgery, University Hospital of Leipzig, 04103 Leipzig, Germany; 4 Department of Surgery, Ruhr-University Bochum, Knappschaftskrankenhaus, 44892 Bochum, Germany; 5 Medical Department I, University Hospital Marienhospital Herne, Ruhr-University Bochum, 44625 Herne, Germany

**Keywords:** kidney transplantation, ischemia reperfusion injury, immunological monitoring, biomarker, graft outcome, graft function

## Abstract

Background: Despite recent advances and refinements in perioperative management of kidney transplantation (KT), early renal graft injury (eRGI) remains a critical problem with serious impairment of graft function as well as short- and long-term outcome. Serial monitoring of peripheral blood innate immune cells might be a useful tool in predicting post-transplant eRGI and graft outcome after KT. Methods: In this prospective study, medical data of 50 consecutive patients undergoing KT at the University Hospital of Leipzig were analyzed starting at the day of KT until day 10 after the transplantation. The main outcome parameter was the occurrence of eRGI and other outcome parameters associated with graft function/outcome. eRGI was defined as graft-related complications and clinical signs of renal IRI (ischemia reperfusion injury), such as acute tubular necrosis (ATN), delayed graft function (DGF), initial nonfunction (INF) and graft rejection within 3 months following KT. Typical innate immune cells including neutrophils, natural killer (NK) cells, monocytes, basophils and dendritic cells (myeloid, plasmacytoid) were measured in all patients in peripheral blood at day 0, 1, 3, 7 and 10 after the transplantation. Receiver operating characteristics (ROC) curves were performed to assess their predictive value for eRGI. Cutoff levels were calculated with the Youden index. Significant diagnostic immunological cutoffs and other prognostic clinical factors were tested in a multivariate logistic regression model. Results: Of the 50 included patients, 23 patients developed eRGI. Mean levels of neutrophils and monocytes were significantly higher on most days in the eRGI group compared to the non-eRGI group after transplantation, whereas a significant decrease in NK cell count, basophil levels and DC counts could be found between baseline and postoperative course. ROC analysis indicated that monocytes levels on POD 7 (AUC: 0.91) and NK cell levels on POD 7 (AUC: 0.92) were highly predictive for eRGI after KT. Multivariable analysis identified recipient age (OR 1.53 (95% CI: 1.003–2.350), *p* = 0.040), recipient body mass index > 25 kg/m^2^ (OR 5.6 (95% CI: 1.36–23.9), *p* = 0.015), recipient cardiovascular disease (OR 8.17 (95% CI: 1.28–52.16), *p* = 0.026), donor age (OR 1.068 (95% CI: 1.011–1.128), *p* = 0.027), <0.010), deceased-donor transplantation (OR 2.18 (95% CI: 1.091–4.112), *p* = 0.027) and cold ischemia time (CIT) of the renal graft (OR 1.005 (95% CI: 1.001–1.01), *p* = 0.019) as clinically relevant prognostic factors associated with increased eRGI following KT. Further, neutrophils > 9.4 × 10^3^/μL on POD 7 (OR 16.1 (95% CI: 1.31–195.6), *p* = 0.031), monocytes > 1150 cells/ul on POD 7 (OR 7.81 (95% CI: 1.97–63.18), *p* = 0.048), NK cells < 125 cells/μL on POD 3 (OR 6.97 (95% CI: 3.81–12.7), *p* < 0.01), basophils < 18.1 cells/μL on POD 10 (OR 3.45 (95% CI: 1.37–12.3), *p* = 0.02) and mDC < 4.7 cells/μL on POD 7 (OR 11.68 (95% CI: 1.85–73.4), *p* < 0.01) were revealed as independent biochemical predictive variables for eRGI after KT. Conclusions: We show that the combined measurement of immunological innate variables (NK cells and monocytes on POD 7) and specific clinical factors such as prolonged CIT, increased donor and recipient age and morbidity together with deceased-donor transplantation were significant and specific predictors of eRGI following KT. We suggest that intensified monitoring of these parameters might be a helpful clinical tool in identifying patients at a higher risk of postoperative complication after KT and may therefore help to detect and—by diligent clinical management—even prevent deteriorated outcome due to IRI and eRGI after KT.

## 1. Introduction

Although kidney transplantation (KT) has become the gold standard in patients with end-stage renal disease (ESRD), effective and safe strategies to prevent allograft loss remain yet to be established [1]. In this context, ischemia reperfusion injury (IRI) is an inevitable event during the initial phase after KT, leading to primary graft dysfunction and alloimmunospecific responses to the graft [1,2]. In the case of KT, clinical consequences of IRI are acute tubular necrosis (ATN) and delayed graft function (DGF), and development of acute rejection episodes, accompanied by interstitial fibrosis progression and chronic allograft nephropathy, which have significant impact on long-term graft function and outcome [2,3].

In recent years, there is mounting evidence that immunological mechanisms are involved in the pathogenesis and inflammatory response of IRI, pointing to the activation of both, the innate and the acquired/adaptive immune system following KT [1,2,3,4,5,6,7]. In the early phase of IRI, inflammation is alloantigen-independent and characterized by activation of classical cells belonging to what we call the innate immune system, such as macrophages, neutrophils, dendritic cells (DC) and lymphocytes (as certain natural killer cells—NK cells). However, also humoral components, mostly complement proteins, cytokines together with endothelial and tubular epithelial cells and adhesions molecules play a major role during the early IRI-associated phase of inflammation [1,2,8]. Recent studies could show that different innate immune cells play an important role in bridging innate and adaptive immunity, which are critical early indicators of innate immunity in the graft and orchestrate inflammation subsequent to IRI (and IRI-related clinical complications such as graft dysfunction, rejection or later graft loss). Interestingly, these cells also seem to play a protective role in the healing process from IRI and also seem to promote immune tolerance after transplantation [4,5,9,10,11,12]. Thus, an improved understanding of these complex mechanisms and interactions (between innate and adaptive immune system in general) may establish the way for more effective treatment strategies of IRI-associated complications after KT.

Although numerous studies in this field have been conducted so far, they do not result in giving precise answers concerning the precise mechanisms and pathophysiologic alterations on which these cells progress or resolve IRI in the early phase after KT, and specifically how to monitor IRI and early graft (dys)function after KT. This insight might help to treat or even prevent IRI-associated complications after KT, aiming at inflammation and mediators of inflammation as potential targets for innovative therapy [2,13,14,15]. However, despite its profound clinical importance, adequate monitoring strategies of innate immune cells and effective management strategies to prevent primary graft dysfunction, reducing graft injury and avoiding subsequent allograft loss, are yet to be established. Detailed insight into these inflammatory responses to IRI may not only lead to enhanced clinical outcomes and prevent graft loss after KT, but may also increase the successful use of marginal grafts and therefore expand the tightly restricted donor organ pool available.

Therefore, the aim of our current study was firstly to evaluate and define the predictive role of different innate immune cells for (immune) monitoring, and early detection and successive interventional strategies for early IRI-associated post-transplant complications in KT recipients. Secondarily, these biomarkers were tested in combination with other well-known clinical parameters as predictors for severity of IRI and graft function together with their possible application as useful biomarkers for clinical practice after KT.

## 2. Methods

### 2.1. Study Population and Study Design

In this prospective study, we enrolled patients who underwent living or deceased-donor kidney transplantation (KT) at the University Hospital of Leipzig between October 2014 and December 2016. Clinical data were obtained from our prospectively collected electronic database and follow-up data were collected until March 2021. A special focus was placed on the immunological monitoring of early renal graft injury (eRGI), specified as IRI-associated post-transplant clinical outcome along with graft function and outcome following KT. Exclusion criteria were age <18 years, multiorgan/combined transplantation, a history of an active infection (including HIV/viral hepatitis), persistent cytomegalovirus, BK infection, autoimmune or hematological disorders, malignant disorders besides nonmelanotic skin cancer within the previous 5 years or a serious injury/major surgery within the preceding month, and missing and incomplete clinical and specific immunological data. All patients provided written informed consent before enrollment and the study protocol was reviewed and approved by the Ethics Committee of the University Hospital of Leipzig (AZ-046-13-28012013 and 046-13-28012013).

### 2.2. Clinical Parameters and Outcome Analysis

Standard demographic, clinicopathological and procedure-related/immunological data were collected and analyzed before, during and after transplantation, and in the follow-up period for each patient: donor and recipient age, gender, body mass index (BMI, weight in kg/height in m^2^), donor cause of death, type of underlying disease leading to end-stage renal disease, number of transplantation, duration of dialysis, time on the waiting list and information on special comorbidities (cardiovascular disease (CVD), diabetes mellitus (IDDM), peripheral artery disease (PAD), arterial hypertension). Peri- and post-transplant data included information on the number of human leukocyte antigen (HLA) -A, B and DR mismatches (0–6), last pretransplant panel reactive antibody (PRA) levels, cold (CIT) and warm ischemia time (WIT) of the graft, duration of operation, Cytomegalovirus (CMV)- state and information on the immunosuppressive therapy. Peri- and postoperative outcome parameters included type of infections (bacterial, viral and fungal), time of hospital stay and average tacrolimus levels during the first 3 months. CIT was defined as the time that the organ spent in cold preservation solution after removal from the donor, while WIT was defined as the time from cross-clamping until cold perfusion, plus the time of implantation (organ out of ice until reperfusion).

As main outcome parameter, the occurrence of early renal graft injury (eRGI) as a compound endpoint was evaluated. This was defined according to previous definitions from the literature as typically graft-related complications and clinically associated outcome parameters and consequences of renal IRI, which was manifest as acute tubular necrosis (ATN) and consequently delayed graft function (DGF)/initial nonfunction (INF) and graft rejection within 3 months following KT [3].

INF was defined as dialysis dependence or creatinine clearance ≤20 mL/min at three months post-transplant, which was not evident in our study population. DGF was defined as the need for dialysis in the first week following transplantation [16]. Rejection episodes were histologically proved. Rejection was defined as any acute T-cell-mediated rejection (TCMR), including “borderline changes” or any C4d-positive antibody-mediated rejection assessed according to the Banff criteria, which were used to guide treatment during the period of the study [17,18,19]. The category “borderline changes” was defined according to Banff 1997 criteria (10–25% interstitial inflammation and at least mild tubulitis) [18]. At our center, “borderline changes” and T-cell mediated rejection (TCMR) (grade I and IIa) were treated by methylprednisolone bolus in parallel with increased baseline immunosuppression, TCMR grades IIb, III and steroid-resistant TCRM by ATG plus increased baseline immunosuppression, and antibody-mediated rejection by plasma exchange and intravenous immunoglobulin.

### 2.3. Measurements of Immunological Parameters

Peripheral blood samples were obtained from each patient at different timepoints (before transplantation and at postoperative days (POD) 1, 3, 7 and 10) to measure the quantity of different innate immune cells, including monocytes, granulocytes, different subsets of dendritic cells (DC) (myeloid dendritic cells (mDC) and plasmacytoid dendritic cells (pDC)) and natural killer (NK) cells, which were correlated with clinical patient data.

### 2.4. Sample Preparation

Blood samples obtained from the 50 patients were drawn in EDTA-treated tubes. The blood samples were gently agitated and split into six different fractions to analyze (1) general immune status; (2) CD4^+^ subpopulations; (3) CD8^+^ subpopulations; (4/5) NK cell subpopulations and activation and (6) dendritic cells. For each fraction, 100 µL of whole blood was incubated with an antibody cocktail specific for the desired cell populations. The detailed composition of the antibody cocktails 1–5 (concentration, clones) was previously described and published elsewhere [20]. For analysis of dendritic cells, we used an antibody cocktail containing of lin 1 FITC, anti-CD11c APC (clone S-HCL-3), anti-CD123 PE (clone 9F5) and anti-HLA-DR PE-Cy5 (clone L243) (all from Becton, Dickinson and Company, BD Biosciences, San Jose, CA, USA). The samples were briefly vortex-mixed and incubated for 15 min at room temperature in the darkness before lysis buffer (BD Biosciences, Heidelberg, Germany) were added for 10 min to lyse erythrocytes. Following centrifugation and washing with PBS (Biochrom, Berlin, Germany), stained cells were fixed with 200 µL PBS containing 1% formaldehyde. Samples were analyzed by flow cytometry immediately thereafter.

### 2.5. Flow Cytometric Analysis and Gating Strategy

For blood sample measurement and data acquisition, a FACS Canto II flow cytometer was used. The cytometer was equipped with three lasers: a 405 nm violet laser, a 488 nm blue laser and a 647 nm red laser, described in detail previously [20]. The data were analyzed using FACSDIVA (Becton, Dickinson and Company, BD Biosciences, San Jose, CA, USA) software.

**Figure 1:**
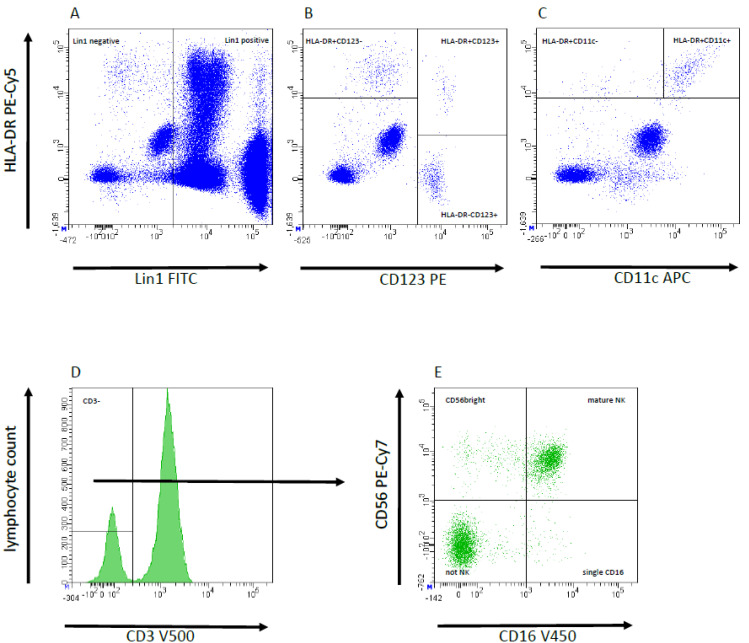
Representative flow cytometry plots from peripheral blood samples to demonstrate gating strategy; typical example illustrating the peripheral blood cell composition with flow cytometric analysis and gating strategy. The antibody cocktail Lin1 includes markers for all leucocyte subtypes, except dendritic cells. Based on lin1-negative cells (**A**) plasmacytoid dendritic cells (pDC) are shown as cells which are HLA-DR^+^CD123^+^ AND HLA-DR^+^CD11c^−^ (**B**,**C**). In contrast, myeloic dendritic cells (mDC) are HLA-DR^+^CD123^−^ AND HLA-DR^+^CD11c^+^ (**B**,**C**). To exclude T cells and NK-T cells, all CD3-cells were separated (**D**). Using antibodies against CD16 and CD56, these cells can be divided CD56 bright, mature NK and single CD16 NK cells. CD16/CD56 double negative were not NK cells (e.g., (**B**) cells), (**E**).

The gating strategy for panel 1–5 was previously described [20] and is illustrated in Figure 1. In short, for NK cells after gating lymphocytes (CD45 APC-H7 vs. SSC), CD3 negative cells were selected (Histogram CD3 V500). Based on that, markers CD16 V450 vs. CD56PE-Cy7 were used to discriminate between CD56bright, mature and single CD16^+^ NK cells. We also measured the expression of Nkp30 PE, Nkp44 Alexa 647, Nkp46 V450, CD57 FITC and CD94 FITC/NKG2D PE in both vs. CD56 PE-Cy7. For analyzing dendritic cells, panel 6 was used, as previously described [20]. First, gating of anti-lin1 FITC vs. HLA-DR PE-Cy5 helps to exclude all CD3^+^/CD14^+^/CD19^+^/CD20^+^/CD56^+^ positive cells and to include dendritic and basophile cells. Based on lin1 neg. cell population, plasmocytoid dendritic cells were identified by gating of CD123 PE vs. HLA-DR PE-Cy5^+^ double positive cell population. In contrast to pDC, myeloid dendritic cells were negative for CD123 PE and positive for CD11c APC.

Finally, absolute cell numbers were calculated by measurement of absolute lymphocytes (derived from particle counter Sysmex XP-300) in relation to relative counts of lymphocytes and subpopulations obtained from the flow cytometer.

### 2.6. Organ Procurement, Transplantation and Immunosuppression

The kidney grafts were procured according to the guidelines provided by Eurotransplant (ET) and transplanted into the iliacal fossa. Deceased-donor kidneys were flushed in situ with cold HTK (histidine–tryptophan–ketoglutarate) solution and explanted. In living-related donation, kidneys were flushed with HTK after donor nephrectomy. For static cold storage, all grafts were immersed in HTK solution at 4 °C [21,22]. The ureter was implanted into the bladder according to the Lich–Gregoir technique using a double J intraureteral splint [23,24].

The initial immunosuppressive therapy in standard patients consisted of a regime of calcineurin inhibitors (CNI) (mostly tacrolimus), mycophenolate mofetil (1000 mg bid) and tapered steroids (prednisolone). In this context, target tacrolimus levels during the first month post-transplant vary according to the different recipient/risk profile between 10 and 15 ng/mL, 5 to 15 ng/mL from the second to the sixth month, and 5 to 10 ng/mL after the sixth month.

Further, patients with specific recipients and/or donor risk profile (high immunological risk, e.g., high PRAs, old donors, living kidney transplantation, retransplantation or ABO-incompatible transplantation, etc.) received an induction therapy with an interleukin-2 receptor antagonist (basiliximab) or antithymocyte globulin (ATG), followed by the regular standard triple maintenance immunosuppression. In this study, none of the patients received an mTOR-based immunosuppressive regime within the first 3 months following transplantation, defining our study period. A rapid steroid-tapering regimen was applied in all our patients, starting with 500 mg methylprednisolone intraoperatively to reach a dose of 25 mg prednisolone at the end of the first week after transplantation. Further reduction intended a daily maintenance dose of 5 mg. Whenever possible, steroids were rapidly withdrawn and discontinued at the end of the first year after transplantation. Subsequent clinical and laboratory patient surveillance (including blood tacrolimus (TAC) levels) vary over time. During the first month post-transplant, the patient is evaluated three times a week, twice a week during the second month, and once a week in the third month. After the third month, evaluations are performed monthly until the first year of follow-up is completed. Because target TAC goals differ through time in clinical practice, we used for further analyses the average tacrolimus levels for 3 months following renal transplantation, in accordance with the study’s primary outcome.

### 2.7. Statistical Analysis

Concerning baseline data, continuous variables are reported as mean values with standard deviation, whereas categorical variables are presented as whole numbers and percentages (%). For comparison between the groups, the appropriate statistical significance test, including Student’s *t*-test, chi-squared test, analysis of variance (ANOVA), Kruskal–Wallis test, and Wilcoxon–Mann–Whitney test were used.

To evaluate the predictive value and prognostic accuracy of innate immune cells in prediction of eRGI, receiver operating characteristic (ROC) curves were generated and the area under the curve (AUC) was calculated. In this context, AUCs ≥ 0.7 were defined as acceptable, AUCs ≥ 0.8 were defined as excellent, and AUCs ≥ 0.9 were defined as exceptional biomarkers [25]. The optimal cutoff was identified using the Youden index (sensitivity +, specificity −1), and sensitivity and specificity were subsequently calculated.

Further, univariate and multivariate logistic regression analyses were used to evaluate the association between independent clinical and paraclinical variables and binary outcomes of eRGI, and separate analysis of DGF and rejection. For the multivariate analyses, we used a backward stepwise regression model including only clinically relevant variables and those presenting *p* < 0.05 in univariate analysis. Results of the regression analyses were presented as odds ratio (OR) with 95% confidence interval (CI) and its corresponding *p*-value. A *p*-value < 0.05 was considered statistically significant. To determine the goodness of fit for the regression model, the Hoshmer–Lemshow test (HLT) was used. The model was considered fit when *p* > 0.05 in HLT. SPSS software (version 28.0; SPSS Inc., Chicago, IL, USA) and Graphpad Prism software (version 9.2.0; Graph-Pad Software Inc., La Jolla, CA, USA) were used for statistical analysis and graphs.

## 3. Results

### 3.1. Baseline Characteristics

Between 2013 and 2015, 90 KT were performed in our department, of which a total of 50 patients could be identified and included in our prospective study. Because of our exclusion criteria, 40 patients were excluded from the study. In detail: in 19 patients a combined pancreas kidney transplantation and in 8 patients multi-organ/combined transplantations were performed, 7 patients were below 18 years and in 6 patients recurrent and (in)active infections, previous malignant disorders or major surgeries within the last month before transplant were present. Twenty-five patients (50%) were male with a mean age of 52.6 ± 14.3 years. Among them, 23 (46%) presented with early renal graft injury (eRGI) and IRI-associated post-transplant complications following KT. The mean follow-up period of the study was 7.2 +/− 3.3 years.

Donor and recipient’s demographic and clinical–pathological characteristics regarding patients with and without eRGI are given in Table 1. In particular, eRGI was more common in donors with a higher BMI (*p* = 0.029) and older age (*p* = 0.01). Patients with eRGI were older (*p* = 0.042), had a higher BMI (*p* = 0.07) and showed a higher presence of cardiovascular disease (*p* = 0.09).

### 3.2. Peri- and Postoperative Outcome

Table 2 shows transplant-related and peri- and postoperative outcome parameters. Mean cold ischemia time (*p* = 0.01) and mean length of hospital stay (*p* < 0.01) were increased in patients with eRGI. Further, bacterial infections (*p* = 0.06), viral infections (*p* = 0.05) and fungal infections (*p* = 0.901) were more common in patients with eRGI, However, average tacrolimus levels were lower in patients with eRGI (*p* = 0.09). As reasons of eRGI (n = 23 Patients) after KT in our study population (n = 50 patients), ATN and delayed graft function was observed in 15 (30%) and 17 (34%) patients, respectively, and/or rejection episodes in 9 (16%) patients. Rejection episodes were classified as borderline changes (n = 3, 33%), acute T-cell mediated rejection (n = 5; 56%) and antibody mediated rejection (n = 1; 11%). The type of induction therapy (with/without ATG) and type of kidney donation (deceased/living donor transplantation) according to occurrence of pooled and compound endpoint eRGI and separately for DGF, rejection, INF and ATN are given in Supplementary Table S1. In short, the application of ATG treatment was comparable between type of kidney donations with evident values of 7% in LD-group, and 11% in the DD-group.

### 3.3. Kinetic Levels of Innate Immune Cells between Both Groups

**Figure 2:**
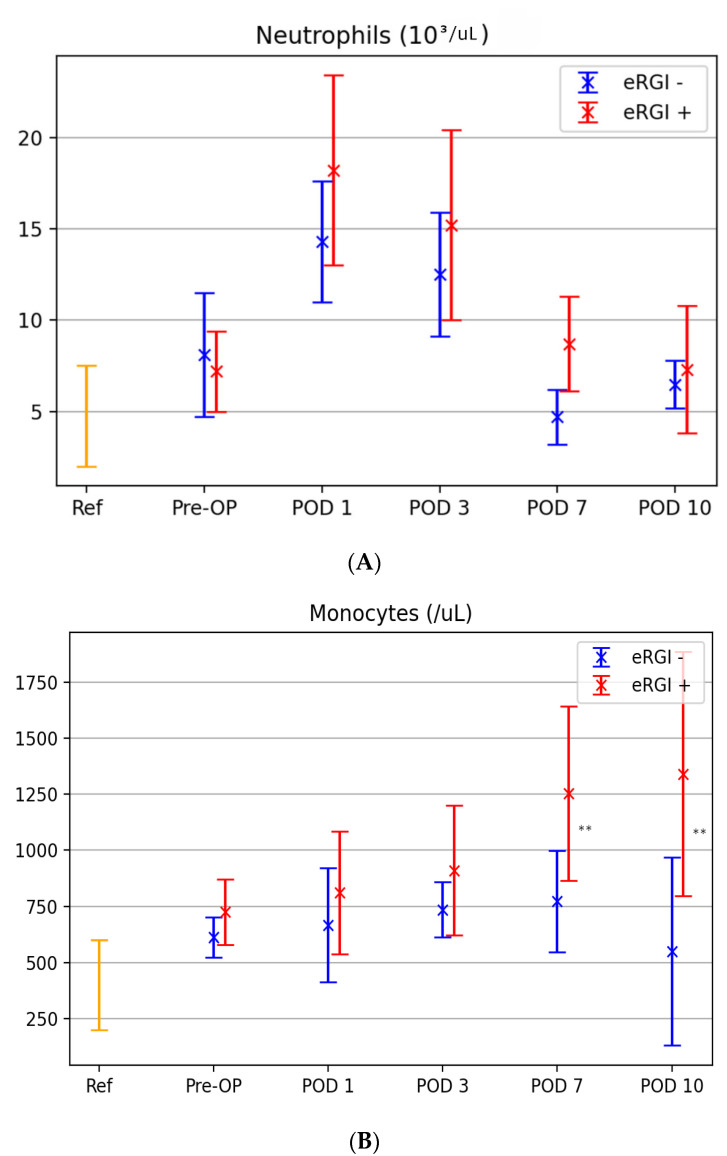

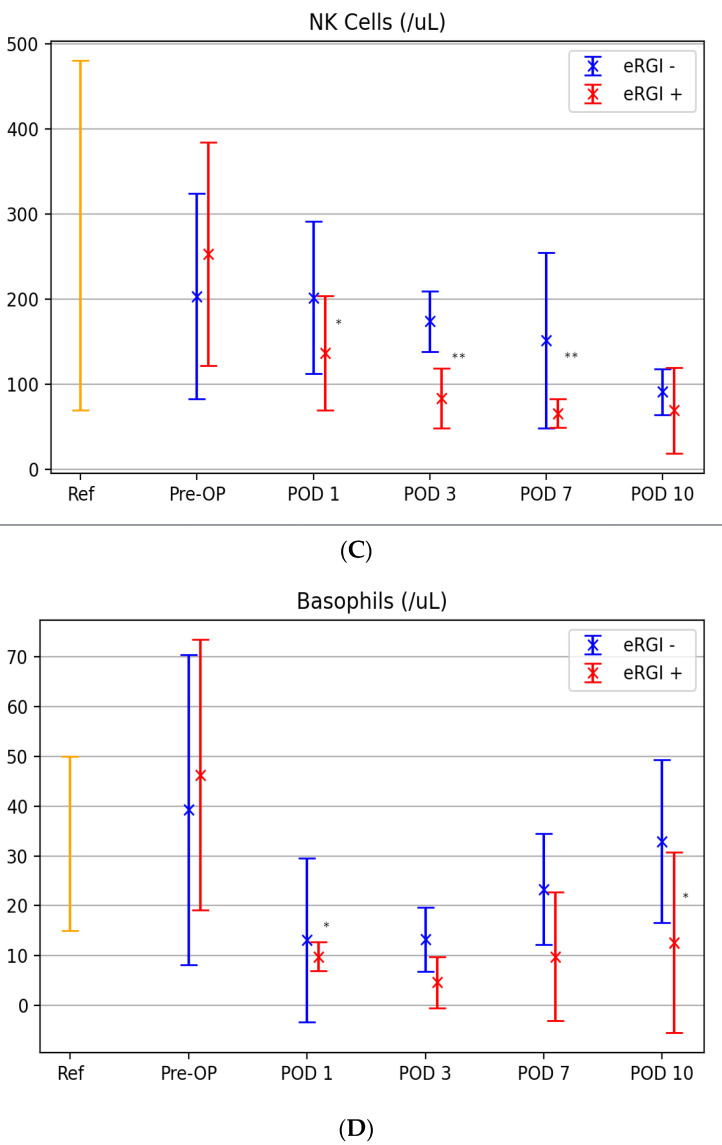

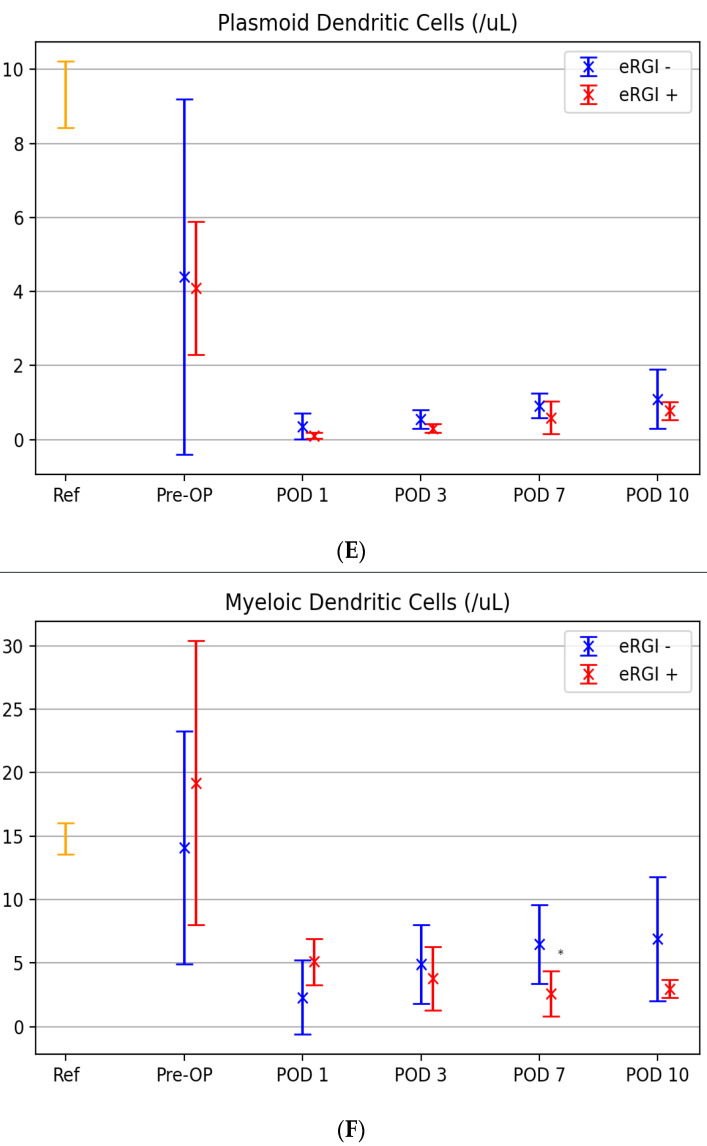
Box plots showing the distribution of neutrophils (**A**), monocytes (**B**), NK cells (**C**), basophils (**D**), mDC (**E**) and pDC (**F**) levels in the eRGI^+^ and eRGI^−^ groups, and standard reference values of innate immune cells established by our laboratory preoperative and on POD 1, 3, 7 and 10 following KT. * *p* < 0.05, ** *p* < 0.01; NS, not significant; POD, postoperative days.

Levels of neutrophils, monocytes, DC subsets (myeloid and plasmacytoid DC), NK cells and basophils were measured at baseline and in the post-transplant period (at POD 1,3, 7 and 10) in both groups (with and without eRGI) (Figure 2). In this context, the standard reference values established by our laboratory are further depicted for better understanding.

Postoperative trends of neutrophils and monoctyes were similar in patients with and without eRGI, showing a peak on POD 1 and POD 2, respectively (Figure 2A,B). Neutrophil levels on POD 1, 3 and 7 and monocyte levels on POD 7 and 10 were significantly higher in patients with eRGI compared to patients without eRGI (all *p* < 0.05). A significant decrease in NK cell count, basophil levels and DC counts was found between baseline and postoperative course in patients with eRGI compared to those without eRGI (Figure 2C–F). In particular, we found the lowest levels of NK cells and myeloid DC on POD 7 (*p* < 0.05), of basophils on POD 3 (*p* < 0.05) and plasmacytoid DC on POD 1 (*p* <0.05).

### 3.4. ROC Analysis for Prediction of Early Renal Graft Injury

**Figure 3:**
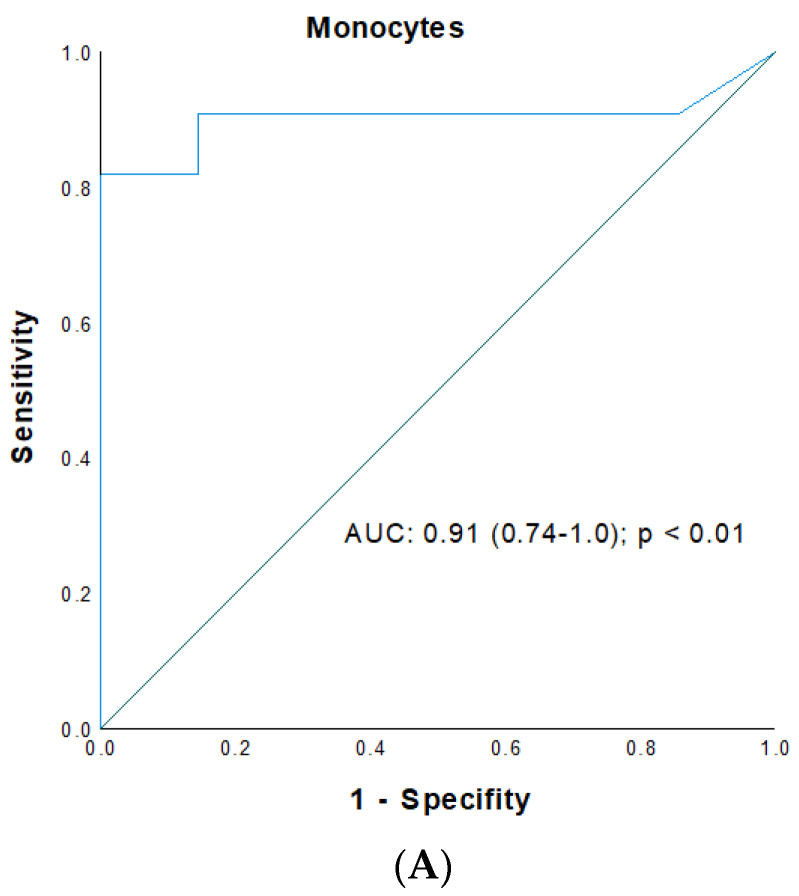

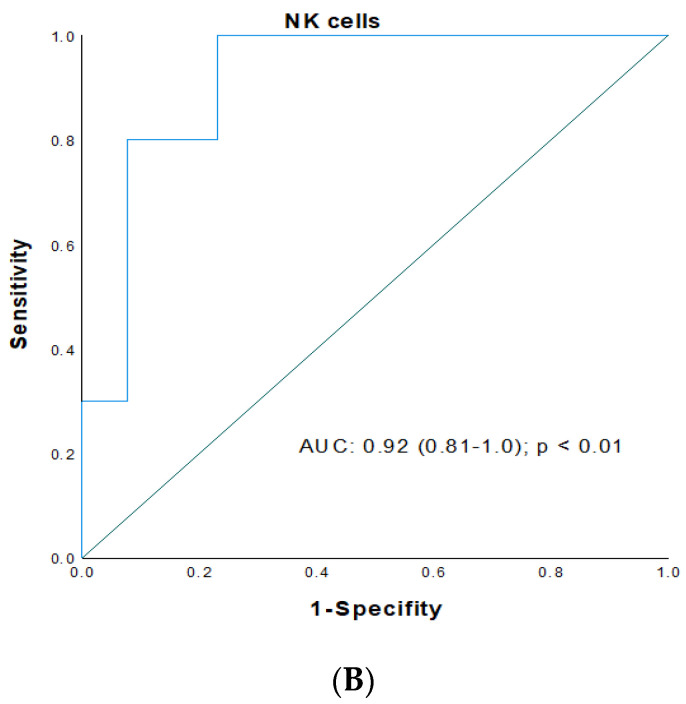
Receiver operating characteristic curve for (**A**) monocyte values on POD 7 and (**B**) NK cell value on POD 7 as predictors for early renal graft injury (eRGI) following renal transplantation.

Cutoff- and AUC values such as specificity and sensitivity of all analyzed innate immune cells, including neutrophils, monocytes, NK cells, DC subsets; and basophils for patients with eRGI (eRGI^+^) and those without (eRGI^−^) were determined using ROC analysis and are listed in Table 3. Concerning eRGI, the diagnostic accuracy—based on the AUCs obtained from ROC plots—of neutrophils (POD 0, 3, 10), monocytes (POD 0, 1 and 3), NK cells (POD 0, 1 and 10), mDC (POD 0, 1, 3 and 7), pDC (all PODs) and basophils (POD 0, 1, 3 and 7) was fair and moderate (AUC < 0.8), whereas neutrophils on POD 1 and 7, monocytes on POD 10, NK cells an POD 3, basophils and mDC on POD 10 were excellent (AUC > 0.8), and monocytes and NK cells on POD 7 were exceptional biomarkers (AUC > 0.9). As depicted in Figure 3, the highest predictive power was observed for monocytes on POD 7 with an AUC of 0.91 (95% CI: 0.74–1.0; *p* < 0.01) with an ideal cutoff of 1150 cells/μL (sensitivity and specificity: 82% and 94%, respectively) (Figure 3A), and an AUC of 0.92 (95% CI: 0.81–1.0; *p* < 0.01) for NK cells on POD 7 with an ideal cutoff of 91 cells/μL (sensitivity and specificity: 99 and 77%, respectively) (Figure 3B) with a valid goodness-of-fit test (monocytes: HLT: *p* = 0.974; chi square: 3.21 and NK cells: chi square: 4.572; *p* = 0.561).

### 3.5. Logistic Regression of IRI

The relationship between patients’ characteristics, intraoperative factors, postoperative immunological markers, and the development of eRGI as the pooled outcome variable following KT is shown in Table 4.

In this context, the following significant factors in univariable analysis with deteriorated prognosis and eRGI were determined: recipient age (*p* < 0.01) and gender (*p* = 0.047), recipient BMI > 25 kg/m^2^ (*p* = 0.027), no pre-emptive transplantation (*p* = 0.027), donor age (*p* = 0.04), deceased-donor transplantation (*p* = 0.004), CIT (*p* = 0.003), operating time (*p* = 0.03), neutrophils on POD 1 and 3 > 12.5 × 10^3^/μL and 14.1 × 10^3^/μL, respectively (*p* = 0.011 and *p* = 0.031), neutrophils on POD 7 > 9.4 × 10^3^/μL (*p* = 0.022), monocytes on POD 7 > 1150 cells/μL (*p* = 0.018), monocytes on POD 10 > 1340 cell/μL (*p* = 0.012), NK cells on POD 3 < 125 cells/μL (*p* = 0.01) and 7 cells/μL < 91 (*p* y 0.01), basophils on POD 10 < 18.1 cells/μL (*p* = 0.019), mDC on day 7 < 4.7 cells/μL (*p* = 0.029) and POD 10 cells/μL < 5.1 (*p* < 0.01).

In multivariable analysis (see Table 4), increased recipient age (OR 1.53 (95% CI: 1.003–2.350), *p* = 0.04), increased recipient BMI (OR 5.6 (95% CI: 1.36–23.9), *p* = 0.015), cardiovascular disease (OR 8.17 (95% CI: 1.28–52.16), *p* = 0.026), increased donor age (OR 1.068 (95% CI: 1.03–1.14), *p* < 0.010), deceased-donor organ transplantation (OR 2.18 (95% CI: 1.091–4.112), *p* = 0.027) and prolonged CIT of the renal graft (OR 1.005 (95% CI: 1.001–1.01), *p* = 0.019) could be identified as independent clinical factors associated with increased eRGI following KT.

Further, the following innate immunological serum markers including neutrophils > 9.4 × 10^3^/μL on POD 7 (OR 16.1 (95% CI: 1.31–195.6), *p* = 0.031), monocytes > 1150 cell/μL on POD 7 (OR 7.81 (95% CI: 1.97–63.18), *p* = 0.048), NK cells < 125 cells/μL on POD 3 (OR 6.97 (95% CI: 3.81–12.7), *p* < 0.01), basophils < 18.1 cells/μL on POD 10 (OR 3.45 (95% CI: 1.37–12.3), *p* = 0.02) and mDC < 4.7 cells/μL on POD 7 (OR 11.68 (95% CI: 1.85–73.4), *p* < 0.01) were revealed as independent predictors for eRGI after KT. HLT showed a valid goodness of fit for the logistic regression model (chi square: 9.11; *p* = 0.12).

Moreover, subanalyses were performed by analyzing the outcome parameters DGF and rejection separately (see Supplementary Materials, Tables S2 and S3).

Concerning the outcome variable for acute rejection, we could demonstrate in a multivariate analysis that increased recipient age (OR 1.11 (95% CI: 1.02–1.21), *p* = 0.018), cardiovascular disease (OR 12.3 (95% CI: 1.9–82.3), *p* < 0.01), increased donor age (OR 1.009 (95% CI: 1.007–1.012), *p* = 0.03), HLA- mismatches > 3 (OR 1.87 (95% CI: 1.15–3.91), *p* = 0.01), delayed graft function (OR 2.49 (95% CI: 1.57–3.93), *p* = 0.03) and bacterial infections (OR 8.5 (95% CI: 1.5–46.7), *p* = 0.019) could be identified as independent clinical factors associated with increased acute rejection following KT. In addition, neutrophils > 9.4 × 10^3^/μL on POD 7 (OR 18.6 (95% CI: 1.49–216.2), *p* = 0.019), monocytes > 1150 cell/μL on POD 7 (OR 14.5 (95% CI: 1.14–187.6), *p* = 0.039), NK cells < 91 cells/μL on POD 7 (OR 10.6 (95% CI: 1.11–110.8), *p* = 0.031), basophils < 6.7 cells/μL on POD 3 (OR 9.43 (95% CI: 4.87–45.8), *p* = 0.049) and mDC < 4.7 cells/μL on POD 7 (OR 22.7 (95% CI: 2.61–198.2), *p* < 0.01) were revealed as independent predictors for acute rejection after KT (Supplementary Table S2).

Furthermore, regarding the outcome variable DGF separately, our subgroup analysis could show in a multivariate analysis that an increased recipient age (OR 1.45 (95% CI: 1.15–1.83), *p* <0.01), a body mass index > 25 kg/m^2^ (OR 1.33 (95%CI: 1.18–1.49), *p* < 0.01), cardiovascular disease (OR 5.28 (95% CI: 1.15–24.3), *p* = 0.029), female donors (OR 5.9 (95% CI: 1.4–25.6), *p* = 0.01), increased donor age (OR 1.13 (95% CI: 1.1.04–1.23), *p* <0.01), deceased kidney transplantation (OR 4.1 (95% CI: 1.09–14.8), *p* = 0.01) and prolonged CIT (OR 1.006 (95%CI: 1.001–1.023), *p* = 0.02) could be identified as independent clinical factors associated with increased DGF following KT. In addition, neutrophils > 9.4 × 10^3^/μL on POD 7 (OR 14.0 (95% CI: 1.54–127.2), *p* = 0.013), monocytes > 1150 cell/μL on POD 7 (OR 23.2 (95% CI: 2.5–311.2), *p* < 0.01), NK cells < 125 cells/μL on POD 3 (OR 5.8 (95% CI: 1.57–29.8), *p* = 0.019), basophils < 18.1 cells/μL on POD 7 (OR 5.21 (95% CI: 1.4–18.9), *p* = 0.021), pDC < 0.6 cells/μL on POD 7 (OR 19.8 (95% CI: 1.9–216.2), *p* < 0.01 and mDC < 4.7 cells/μL on POD 7 (OR 10.5 (95% CI: 1.01–108.5), *p* = 0.018) were revealed as independent predictors for increased DGF after KT (Supplementary Table S3).

## 4. Discussion

IRI and subsequent early graft dysfunction remain a relevant clinical problem affecting all organs utilized for transplantation and play an important role for short- and long-term graft function and outcome [1,2,3,5]. Mounting evidence suggests that IRI-induced organ damage is linked to alloimmune-independent and -dependent immune responses in a complex interplay of innate and adaptive immunity [2,5]. Well-considered monitoring of specific innate serological biomarkers could therefore be a helpful tool for early detection and diagnosis of IRI-induced graft damage and also for finding targeted treatment choices by addressing signal pathways of innate and adaptive immunity leading to amelioration of IRI-induced organ damage. Thereby, the main clinical goal should be the prediction of the individual’s risk of allograft injury and ischemic damage together with outcome prediction after transplantation, resulting in individualized treatment approaches and outcome improvement after solid organ transplantation [2,14,26,27].

Therefore, in our current study, we used a panel of standardized, commonly available, and relatively inexpensively measured innate immune biomarkers in the peripheral blood for their value in predicting early renal graft injury and graft function/outcome in patients following KT.

Hereby, monocytes and NK cells on POD 7 showed the most intriguing results with exceptional predictive values (AUC > 0.9, respectively), demonstrating a high predictive accuracy for detection of early renal graft injury and graft function. According to the findings of our study, the assessment of specific innate biomarkers combined with other pre-, intra- and postoperative donor- and recipient-related, and transplant-associated clinical and paraclinical factors (in particular, recipient age, recipient BMI > 25, cardiovascular disease, donor age, type of transplant, CIT of the graft) may present helpful insights, in order to identify patients at high risk of eRGI and to predict long-term graft function and outcome in this vulnerable patient population. These new insights of combined clinical and immunological prediction aspects could also be observed not only for the pooled outcome variable eRGI, but as well as for the separate subgroup analyses for DGF and rejection (see Supplementary Tables S1–S3).

Previous studies in the transplant setting could show that neutrophils are multipotent players, which are able to activate both innate and adaptive immune cells, and share—together with monocytes—the role of first responders in reaching the allograft during the inflammatory process triggered by IRI [5,28]. Thus, during this IRI-induced inflammatory process, transient elevation and activation of neutrophils, the largest circulating fraction of leukocytes, could be observed in the early postoperative period after reperfusion in KT [5,28]. Although the presence of neutrophils within the graft is usually linked to bacterial infection, one should consider their crucial role as markers of either rejection processes or—especially when they present together with T cells—as potential makers of nonalloimmune and alloimmune responses following inflammatory processes [5,29].

In this context, animal models could previously show the detrimental role of neutrophils in the pathogenesis of acute kidney injury and IRI following KT, with promising results by pharmacological prevention of neutrophil migration and invasion of graft tissue, and subsequent attenuation of acute rejection [1,2]. However, these promising results could not entirely be confirmed in humans. Although a phase I study of a clinical trial investigating the use of anti-ICAM-1 monoclonal antibody in 18 recipients of deceased-donor renal transplants showed lower rates of DGF, a randomized controlled trial failed to show beneficial effects of ICAM-1 blocking on the rate of DGF [1,2,30,31]. Of interest, a recent study on immune monitoring after liver transplantation could demonstrate that high levels of neutrophils in peripheral blood on POD 7 are correlated well with infection and rejection rates, indicating typical clinical consequences of IRI [14]. These findings are in accordance with previous studies in the setting of kidney transplantation, showing that neutrophils may be helpful in discrimination and detection of acute rejection [32]. Our current study is in line with these previous findings, confirming the important role of neutrophils in IRI-induced organ damage. Moreover, we could show for the first time that neutrophil levels on POD 1 and 7 show excellent predictive correlations (>0.8 AUC) for eRGI and graft-related complications after KTA. Additionally, neutrophil levels with a cutoff value of >9.4 × 10^3^/μL on POD 7 could be identified as an independent prognostic parameter in multivariate logistic regression analysis for eRGI following KTA. Of interest, neutrophil levels on POD 3 and 10 had only an acceptable predictive accuracy (<0.8 AUC) for eRGI in our current study.

Regarding our findings on monocyte levels, their high values, particularly on POD 7, in patients who developed IRI and IRI-related clinical consequences, as well as their excellent prognostic predictive value, are consistent with their crucial role as acute inflammatory, phagocytosis and immune regulation markers of both the innate and adaptive immune system. In this context, results of previous clinical studies suggest that monocytes—and their different subsets—based on different phenotypes and cell function (e.g., expression of membrane antigens CD14^+^, 16^+^, 47^+^, 163^+^ or HLA-DR) play a key role in IRI and IRI-related clinical complications, particularly in inducing allograft rejection and early graft dysfunction leading to worse graft outcome and function [5,33,34]. However, the majority of these findings were gathered from graft biopsies, which represent highly invasive and risky procedures that involve potential complications for graft and patient, and are not easily accessible. However, the predictive value of blood monocytes during proinflammatory conditions such as IRI following solid organ transplantation, is far less clearly described [5,35,36,37]. A clinical immune monitoring study showed a promising role of circulating CD46 positive monocytes predicting complications in the first postoperative days (especially on POD 7) following liver transplantation [14]. In the field of kidney transplantation, previous studies by Van den Bosch et al. and Sablik et al. could recently prove that higher (pretransplant) blood levels of CD16 monocytes are associated with a significantly higher rejection risk (acute and/or chronic) and a shorter rejection-free survival period [38,39]. This is in accordance with another study showing that CD163 overexpression on circulating monocytes was highly useful in prediction of transplant function after KT, and seemed to correlate significantly with serum creatine values at one year after the transplantation [40]. On the other hand, a study monitoring HLA-DR positive monocytes in KT recipients documented a decrease in the number of these monocytes two weeks after transplantation, leading to conflicting results in this field. However, changes in HLA-DR expression were not associated with acute rejection or CMV infection [41]. Our current study adds significant insight into this intriguing field of research, indicating an increase in monocytes in peripheral blood on POD 7 as a highly predictive marker for early renal graft injury in the context of IRI-induced organ damage. Thus, we here show for the first time an easily accessible and noninvasive method for immune-based detection of increased risk of eRGI and subsequent graft failure after KT. Based on the analyses of our data, a very special role may be assigned to an increase in monocyte count in peripheral blood, indicating blood monocytes in the early time course after KT as highly useful biomarkers in clinical practice as an early appropriate (diagnostical, prognostical and therapeutical) tool to predict early renal graft injury, determine the prognosis of kidney function after transplantation and to identify patients at higher risk for early graft loss. Additionally, the results of our current study might be helpful in the future to investigate the value of monocyte subsets in assessment of risk stratification and personalized optimization of immunosuppressive treatment as a cornerstone of pretransplant clinical workup of renal transplant recipients.

There is growing evidence indicating that NK cells may affect allograft outcomes following solid organ transplantation [42]. However, the exact impact, activation mechanisms and function of NK cells in IRI and IRI-associated clinical complications, for instance in IRI-induced allograft rejection, delayed graft function or infections following KT, remain controversial and are not fully understood. In previous studies, NK cells also have been demonstrated to serve a paradox role in both, allograft acceptance and dysfunction after solid organ transplantation, which was due to their effect on the immune pathways involved in both allograft tolerance as well as rejection [43,44]. In the past, Jung et al. could show that patients with antibody-mediated rejection had high densities of NK cells, mainly CD56^+^/57^+^ infiltrate subsets, in the allograft [45]. In renal allografts, this NK cell infiltration was significantly and independently associated with increased graft failure. Other reports show that increased rates of NK cell subsets (CD16^+^, CD56^+^) were indicative of antibody-mediated cytoxicity in chronic rejection or promoted development of acute transplant rejection [39,43].

Furthermore, NK cells as part of the innate immune system seem to play an important role in the immediate protection against infections after solid organ transplantation [46]. In this context, a recent study from Australia could show that reduced NK cell function compared to cell number was a good independent predictor of severe infection following KT [47]. In contrast, other reports in liver and renal transplant patients also show that NK cell numbers were good and independent predictors of opportunistic infections, for instance CMV or invasive fungal infections [48,49]. These findings are clearly supported by the results of our current study, which showed that reduced levels of NK cells in the postoperative course (especially on POD 7) were strong and independent predictors of early renal graft dysfunction and injury.

Although DC have been shown to play crucial key roles in many (renal) diseases and are known as early initiators of innate immunity, little is known about their role in KT and IRI-induced complications with subsequent chronic inflammatory immune reactions leading to DGF or graft rejection [5,10,50]. Based on their developmental, phenotypical and functional features, DCs can be categorized into several different subtypes, of which, in particular, pDC (plasmocytoid dendritic cells) and mDC (myeloid dendritic cells) were examined in our current study. In this context, rejection-associated DCs promote acute or chronic injury in renal grafts, while tolerogenic DCs suppress the overwhelming inflammatory response, thereby preventing damage to renal graft function [10,51]. Previous reports have demonstrated a significant reduction in DC levels following renal transplantation in the first postoperative days, an effect more pronounced for the pDC subset [52,53,54]. These dynamic and circulatory changes are opposite to that of mDC following renal transplantation. Accordingly, previous research in the transplant setting could show that higher levels of DC—especially myeloid DC—in the postoperative course were associated with higher rates of rejection episodes [14,51,54,55,56]. mDCs could also be observed more often in patients with DGF and ATN [9,57]. Sun et al. could recently show that mDC levels before transplantation were independently associated with the occurrence of infections after transplantation, mainly concerning CMV-related infections, and that there was a significant correlation between mDCs and patient survival following renal transplant [58]. Our current study is in line with these interesting findings, adding intriguing insight in the often overlooked DC species and their specific and important role—in particular, myeloid DCs—as independent and excellent prognostic predictors for early renal graft injury on POD 7 after KT.

Nevertheless, even if the immune response and effects of IRI were effectively controlled in the acute phase after transplantation and IRI-induced clinical complications were successfully overcome or avoided, there is still a high possibility that subsequent immune response mediated by chronically activated antibodies will trigger chronic rejection and lead to graft failure in the latter phase after kidney transplantation [9,51].

Within this frame, many studies in the transplant setting started to use immune cells to regulate and manipulate immune responses in order to prevent IRI and IRI-related complications and to prolong allograft survival and function as well as to promote transplant tolerance [2,26,50,59,60]. Consequently, considerable interest has developed in the therapeutical potential of innate and adaptive immune cells, with in situ targeting of (adaptive/innate) (tolerogenic) immune cells (e.g., DC) or by application and (ex vivo) generation of (tolerogenic) innate (including DCreg and Mreg) and adaptive (Treg) immune cells of donor or recipient origin [26,51,59,60]. So far, these treatment modalities have already shown encouraging results in experimental models and are currently being evaluated in clinical trials. In this context, one recent study coordinated some early phase 1 and 2 clinical trials that examined the therapeutic effects of DCreg, Treg and/or Mreg application in renal and liver transplant recipients [60]. First promising results could show that regulatory cell therapy is achievable and safe in living-donor kidney transplant recipients, and is associated with fewer infectious complications, but similar rejection rates in the first year [61].

So far, little is known about the effect of basophils in inflammatory response associated with IRI and IRI-associated clinical complications following solid organ, and in particular, renal transplantation. Recently, there is increasing evidence implicating basophils in a wide range of processes during IRI of hepatic, pulmonary, myocardial, cerebral, renal and intestinal organs [62,63,64]. In this context, there are currently promising approaches in development of specific basophil-targeted therapeutics to protect against IRI in the field of hepatic and cardio pulmonal organ systems [62,64,65,66]. In the special field of transplantation, previous studies could show that there is a clear relationship between occurrence of basophils and graft rejection as well as DGF in renal and skin transplantation [67,68,69]. This is in line with findings of our current study, where we could demonstrate that in our patient collective a basophil level of <18.1 cells/μL on POD 10 was not only an excellent (AUC > 0.8), but also an independent prognostic marker in the prediction of IRI- related clinical consequences and worse outcome following KT.

The effects of antirejection drugs on innate immune cells is profound and it is not yet fully understood how these different methods and consecutive immunological changes both of induction (mainly ATG) and maintenance immunosuppressive therapy influence the different peripheral blood mononuclear cell subpopulations in the early as well as late phase of KT. Based on our small patient population and the even smaller number of given induction therapeutics (ATG) per intention group, no correct and adequate statistical conclusions can be drawn in the absence of statistical power. Our data only show a homogeneous and comparable distribution and use in the application of ATG per living- (7%) and dead-donor recipients (11%) after kidney transplantation (see also Table S1), and further clinical studies are certainly required to gain deeper insights in the intriguing topic of the influence of induction therapeutics and maintenance immunosuppressive therapy on the innate immune response.

Regarding current and previous literature, Sekerkova et al. recently demonstrated a modulation of monocyte subpopulations (CD14^+^CD16^+^ and CD14^+^/CD163^+^), which was partially affected by the use of different immunosuppressive induction therapy regimes. Herein, the proportions of peripheral CD14^+^CD16^+^ monocytes were downregulated immediately after the kidney transplantation in patients without induction therapy and ATG, whereas basiliximab treatment partially attenuated this trend. This transient downregulation of the CD14^+^CD16^+^ subpopulation was adjusted to basal values in two months. On the other hand, the proportions of CD14^+^CD163^+^ monocytes were transiently upregulated in the early phase after the kidney transplantation and remained higher during the first month in most patients. In ATG treated patients, the expansion of CD14^+^CD163^+^ monocytes was delayed but their upregulation lasted longer [33]. These findings are in line with our results in monocytes and DC (data not shown), where we observed a strong decrease in monocytes in the first phase under ATG, whereas patients without induction therapy and basiliximab showed an attenuated decrease. In a similar study, Kho et al. recently showed that in renal transplant recipients, receiving induction therapy with ATG (in a dose dependent manner), NK cells were significantly downregulated immediately after transplantation compared to patients without induction therapy [70]. These findings could be underlined by Krepsova et al., who could show that NK cells in patients with ATG were significantly downregulated on day 7 post-transplant compared to moderate downregulation in patients with basiliximab or without induction therapy [71]. However, patients without induction therapy reached their pretransplant baseline values already at day 14 post-transplant. Concerning DC, a previous study by Womer et al. examined the effects of renal transplantation on peripheral blood dendritic cells. A dramatic decrease was observed post-transplant in both subsets, with a greater reduction in pDC levels. Subgroup analysis revealed significantly greater mDC reduction in RT recipients receiving antilymphocyte therapy, with preferential binding of antibody preparation to this subset. Samples from later timepoints revealed a gradual return of DC levels back to pretransplant values concurrent with overall reduction in immunosuppression [72]. Further studies are surely required to further evaluate how the levels of innate (and adaptive) immune cells are influenced by the degree and different ways of immunosuppressive regimes, and for a better understanding regarding the role of these cells in the following allotransplant response. Furthermore, adequate monitoring of these innate immune cells may represent a novel and sexy strategy to assess the degree of immunosuppression in individual transplant recipients, which may be predictive of important outcomes such rejection, graft loss or infection.

Although our current study shows unique and promising results, several limitations are important to discuss.

Firstly, despite the prospective design of our current study, due to the low number of included patients, the main results and statistical findings must be critically considered and interpreted with caution before extrapolating our results to common clinical practice. Therefore, we propose as a future study approach to conduct a large multicenter clinical observational trial with immune monitoring before and after KT to gain more detailed insight into this intriguing topic with, for instance, generation of multivariate logistic regression analyses with more statistical power and higher number of patients per intention group to confirm the current findings of our study.

Secondly, although in our current study a variety of the most common circulating serological markers of innate immunity (regarding the monitoring of KT-induced IRI and IRI-related clinical consequences) was evaluated and analyzed, no quantification and testing of regulatory innate immune cells ((i.e., regulatory dendritic cells (Treg) or regulatory macrophages (Mreg)/regulatory dendritic cells (DCreg)) was performed. In addition, no detailed analysis and measurement of function and relationship of the humoral components (mostly complement proteins, cytokine or cell adhesion molecules) of the innate immune system were undertaken.

Thirdly, the results of our study must certainly be viewed critically, since we have investigated an immunologically and functionally different group of donor organs (living and deceased donors). On the other hand, although the immunosuppressive maintenance therapy of our recipients was identical, the induction therapy (ATG versus basxiliximab versus none) was different, which can also have an influence on the course of the innate immune cells. Therefore, larger multicenter studies with a higher number of patients per intention group and the identical donor/risk profile would certainly be useful to investigate the distinct kinetic course, value and influence of innate immune cells in the early allotransplant response, and to further investigate the influence of different IS regimes and degrees on the course of these immune cells and the resulting early transplant function and outcome. In this context, it could be useful in the future to routinely monitor and evaluate innate immune cell function and activity in the context of specific algorithms to personalize immunosuppressive regimes.

Therefore, in the future, larger prospective multicenter (omic-associated) studies are needed to firstly examine the role and relationship of these new and promising innate immune parameters in both the graft and surrounding lymphoid tissues and in the peripheral blood for monitoring and prevention of IRI and IRI-related clinical outcome. Moreover, these studies might help to generate clinical protocols for the translation of regulatory-based immune cell therapy to clinical practice for treatment of IRI and to prolong graft function and outcome.

## 5. Conclusions

Accurate and, especially, early identification and monitoring of patients with increased risk for IRI and IRI-related clinical complications following KT is elementary to guide clinical decision-making and to select those patients who might benefit from novel prophylactic treatment. Our current study gives first insights that some innate immune cells (including neutrophils, monocytes and NK cells) on different days in the postoperative course after KT were excellent and prognostic predictive markers for the detection and monitoring of IRI, and subsequently IRI-associated early graft dysfunction and clinical complications. The resulting risk groups according to our proposed marker cutoff levels allow for improved risk stratification and better risk-adapted monitoring for perioperative complications following KT compared to established clinical and paraclinical criteria such as CIT, recipient characteristics (e.g., BMI, CVD) or special donor variables. Therefore, the future treatment of IRI-induced organ damage may lie in targeting the inflammatory responses of IRI by aiming at pathways of both the innate and the acquired immune system, possibly even during the process of graft and recipient conditioning before and during the transplantation, and in the early and late postoperative period. With these multimodal approaches, perhaps in combination with techniques already applied, such as ischemic or volatile anesthetic-induced preconditioning, we might succeed in the future to reduce the detrimental effects of IRI and thereby improve long-term graft outcome after kidney transplantation. These novel therapeutic possibilities in combination with strong diagnostical biomarkers for the prediction of early renal graft dysfunction may be analyzed in future studies, evaluating new ways to improve impaired microcirculation following renal transplantation and to extend survival and function of transplanted organs.

## Figures and Tables

**Table 1:** Baseline transplant characteristics of recipient and donor according to the status of early renal graft injury (eRGI).

Variables	Total (n = 50 Patients)	eRGI^−^ (n = 27 Patients)	eRGI^+^ (n = 23 Patients)	*p*-Value
**Donor**				
Age, years	54.2 ± 15.9	51.4 ± 15.4	59.9 ± 16.5	0.01
Gender, male/female	25 (50)/25 (50)	12 (44)/15 (56)	13 (57)/10 (43)	0.395
BMI, kg/m^2^	26.2 ± 4.3	25.3 ± 2.9	27.4 ± 4.8	0.029
Hypertension (yes/no)	18 (36)/32 (64)	7 (26)/20 (74)	11 (48)/12 (52)	0.108
Diabetes mellitus (yes/no)	3 (6)/47 (94)	1 (4)/26 (96)	2 (9)/21 (91)	0.459
**Recipient**				
Age, years	52.62 ± 14.3	48.85 ± 14.9	56.1 ± 13.3	0.042
Gender, male/female	34 (68)/16 (32)	15 (56)/12 (44)	19 (83)/4 (17)	0.09
BMI, kg/m^2^	25.5 ± 3.4	24.7 ± 3.4	26.1 ± 3.5	0.07
Diabetes	7 (14)	3 (6)	4 (8)	0.624
Peripheral Arterial Disease	3 (6)	2 (4)	1 (2)	0.650
Previous Dialysis	40 (80)	21 (78)	19 (83)	0.670
Dialysis modality				
Hemodialysis/peritoneal	34 (85)/6 (15)	17 (81)/4 (19)	17 (90)/2 (10)	0.451
Duration of dialysis, months	59.9 ± 7.9	34.9 ± 5.9	30.1 ± 3.8	0.175
Waiting time, months	28.04 ± 30.3	21.9 ± 28.2	24.7 ± 31.9	0.145
Cardiovascular disease	10 (20)	3 (6)	7 (14)	0.09
Type of Donation				
Deceased Donor	35 (70)	15 (66)	20 (87)	0.018
Living Donation	15 (30)	12 (44)	3 (13)	
Original renal disease				0.686
Glomerulonephritis	18 (36)	8 (16)	10 (20)	
Diabetes mellitus	5 (10)	2 (4)	3 (6)	
Vascular/hypertension	10 (20)	7 (14)	3 (6)	
Zystic	7 (14)	4 (8)	3 (6)	
Other	10 (20)	6 (12)	4 (8)	
Number of transplants				0.145
First/Retransplant	43(86)/7 (14)	25 (93)/2 (7)	18 (78)/5 (22)	
HLA- mismatches > 3	26 (52)	13 (48)	13 (56)	0.554
Panel reactive antibodies	8.1 ± 16.9	4.1 ± 8.1	12.8 ± 22.8	0.06

**Table 2:** Transplant-related and peri- and postoperative outcome for patients with early renal graft injury (eRGI) and those without, following kidney transplantation.

Variables	Total (n = 50 Patients)	eRGI^−^ (n = 27 Patients)	eRGI^+^ (n = 23 Patients)	*p*-Value
Warm ischemia time, minutes	43.2 ± 8.1	42.1 ± 9.8	44.6 ± 6.1	0.495
Cold ischemia time, minutes	511 ± 253	411 ± 198	612 ± 212	0.01
Operating time, minutes	206 ± 59.5	196.8 ± 49.1	219 ± 70.3	0.211
Hospital stay, days	27 ± 19.3	23 +/− 16.2	32 +/− 21.5	<0.01
Induction therapy (ATG/IL-2 RA/none)	5 (10)/27 (54)/18 (36)	2 (4)/14 (28)/11 (22)	3 (6)/13 (26)/7 (14)	0.667
CMV-state; D+/R−	18 (36)	11 (22)	7 (14)	0.449
Average tacrolimus levels, ng/ml	8.19 ± 4.6	8.3 ± 3.4	7.99 ± 5.2	0.08
Bacterial infections	17 (34)	6 (19)	11 (43)	0.06
Viral infections	13 (26)	4 (14)	9 (39)	0.05
Fungal infections	2 (4%)	1 (3.7)	1 (4.3)	0.901

**Table 3:** ROC Analysis of different innate immunological markers on different pre- and postoperative days for predicting early renal graft injury (eRGI) following kidney transplantation.

**Days**	**Neutrophils**				**Monocytes**				**NK Cells**			
	**Cutoff**	**AUC (CI)**	**Sensitivity/** **Specificity**	***p*-Value**	**Cutoff**	**AUC (CI)**	**Sensitivity/** **Specificity**	***p*-Value**	**Cutoff**	**AUC (CI)**	**Sensitivity/** **Specificity**	***p*-Value**
0	7.7	0.43 (0.38–0.79)	56/62	0.165	550	0.64 (0.34–0.94)	85/75	0.335	129	0.60 (0.34–0.85)	80/39	0.439
1	12.5	0.83 (0.66–0.99)	67/88	0.011	725	0.75 (0.51–0.98)	72/78	0.151	177	0.72 (0.49–0.95)	76/81	0.078
3	14.1	0.79 (0.61–0.99)	75/90	0.028	850	0.66 (0.41–0.90)	78/62	0.236	125	0.84 (0.66–1.0)	91/73	<0.01
7	9.4	0.89 (0.72–0.99)	83/86	<0.01	1150	0.91 (0.74–1.0)	82/94	<0.01	91	0.92 (0.81–1.0)	99/77	<0.01
10	8.2	0.64 (0.59–0.79)	54/78	0.456	1340	0.89 (0.71–0.98)	78/91	<0.01	66	0.67 (0.42–0.92)	62/91	0.181
**Days**	**Basophils**				**mDC**				**pDC**			
	**Cutoff**	**AUC (CI)**	**Sensitivity/** **Specificity**	***p*-Value**	**Cutoff**	**AUC (CI)**	**Sensitivity/** **Specificity**	***p*-Value**	**Cutoff**	**AUC (CI)**	**Sensitivity/** **Specificity%**	***p*-Value**
0	11.7	0.54 (0.21–0.88)	83/42	0.775	9.8	0.51 (0.16–0.83)	83/49	0.865	3.89	0.52 (0.18–0.87)	83/43	0.886
1	7.15	0.79 (0.54–1.0)	67/75	0.071	5.5	0.74 (0.13–0.89)	66/88	0.123	0.76	0.46 (0.25–0.58)	93/51	0.247
3	6.73	0.69 (041–0.98)	86/59	0.214	3.8	0.63 (0.46–0.95)	62/73	0.465	0.26	0.61 (0.48–0.74)	58/49	0.257
7	12.2	0.77 (0.49–0.97)	71/88	0.08	4.7	0.80 (0.55–0.92.)	93/82	0.032	0.55	0.77 (0.56–0.98)	86/57	0.09
10	18.16	0.85 (0.63–0.99)	83/89	0.024	5.1	0.89 (0.71–0.99)	99/78	0.013	0.95	0.69 (0.45–0.94)	88/68	0.127

**Abbreviations:** AUC, area under the curve; CI, confidence interval; mDC, myeloid dendritic cell; pDC, plasmacytoid dendritic cell.

**Table 4:** Uni- and multivariate logistic regression analysis of clinical and immunological predictors for early renal graft injury (eRGI) after kidney transplantation.

Variables	UVA	MVA	*p*-Value
	*p*-Value	OR (95% CI)	
**Recipient factors**			
Recipient age, years	<0.01	1.53 (1.003–2.350)	0.040
Recipient gender (female versus male)	0.047	NS	
Recipient BMI, <25 versus >25	0.027	5.6 (1.36–23.9)	0.015
Pre-emptive transplantation (no versus yes)	0.027		
Recipient cardiovascular disease (yes versus no)	<0.01	8.17 (1.28–52.16)	0.026
**Donor factors**			
Donor age, years	0.004	1.068 (1.011–1.128)	0.012
Donor BMI, per 5 kg/m^2^	0.08	NS	
Donor type—DD versus LD	0.004	2.18 (1.091–4.112	0.027
**Transplant-related factors**			
Cold ischemia time	0.003	1.005 (1.001–1.01)	0.019
HLA- mismatches >3 versus <3	0.08		
Operating time	0.03	NS	
Number of transplant (re versus first)	0.110		
Average tacrolimus levels; > versus < 8 ng/mL	0.07		
Fungal infections	0.908		
Viral infections	0.06		
Bacterial infections	0.05		
**Immunological parameters**			
Neutrophils POD 1; >12.5 × 10^3^/μL	0.011	NS	
Neutrophils POD 3; >14.1 × 10^3^/μL	0.031	NS	
Neutrophils POD 7; >9.4 × 10^3^/μL	0.022	16.1 (1.31–195.6)	0.031
Monocytes POD 7; >1150 cells/μL	0.018	7.81 (1.97–63.18)	0.048
Monocytes POD 10; >1340 cells/μL	0.012	NS	
NK cells POD 3; <125 cells/μL	<0.01	6.97 (3.81–12.7)	<0.01
NK cells POD 7; <91 cells/μL	<0.01	NS	
Basophils POD 10; <18.1 cells/μL	0.019	3.45 (1.37–12.3)	0.024
mDC POD 7; <4.7 cells/μL	0.029	11.68 (1.85–73.4)	<0.01
mDC POD 10; <5.1 cells/μL	<0.01	NS	

**Following variables were tested in univariate analysis but failed to show significancy**: warm ischemia time, induction therapy (yes versus no), donor cerebrovascular disease as cause of death (yes versus no); donor gender (female versus male); recipient comorbidities (diabetes mellitus; peripheral arterial disease); time on dialysis pretransplant; immunological factors: neutrophils POD 0 and 10; monocytes POD 0, 1 and 3; NK cells on POD 0, 1 and 10; basophils on POD 0,1,3, and 7; pDC on POD 0, 1, 3, 7 and 10. **Abbreviations:** UVA, univariate analysis; MVA, multivariate analysis; OR, odds ratio; CI, confidence interval; BMI, body mass index; NS, not significant; LD, living donor; DD, deceased donor; mDC, myeloid dendritic cells; pDC, plasmocytoid dendritic cells; POD, postoperative days.

## Data Availability

Our database contains highly sensitive data that may reveal clinical and personnel information about our patients and lead to their identification. Therefore, according to organizational restrictions and regulations, these data cannot be made publicly available. However, the datasets used and/or analyzed in the current study are available from the corresponding author upon reasonable request.

## Supplementary Materials

The following supporting information can be downloaded at: https://www.mdpi.com/article/10.3390/jcm11206148/s1, Table S1: Frequencies type of induction therapy (with/without ATG) and type of kidney transplantation (living/deceased donor kidney transplantation) with regard to the occurrence of the component endpoint itself (i.e. early renal graft injury (eRGI)), and each single component of the combined endpoint: delayed graft function (DGF), rejection, acute tubular necrosis (ATN), and initial non function (INF). Table S2: Uni- and multivariate logistic regression analysis of clinical and immunological predictors for acute (treated) rejection after kidney transplantation. Table S3: Uni- and multivariate logistic regression analysis of clinical and immunological predictors for delayed graft function following kidney transplantation.

## Abbreviations

ANOVA	analysis of variance
ATG	antithymocyte globulin
ATN	acute tubular necrosis
AUC	are under the curve
BMI	body mass index
CI	confidence interval
CIT	cold ischemia time
CMV	cytomegalovirus
CVD	cardiovascular disease
DC	dendritic cell
DCreg	regulatory dendritic cells
DGF	delayed graft function
eRGI	early renal graft in jury
ET	Eurotransplant
HLA	human leukocyte antigen
HLT	Hoshmer–Lemshow test
HTK	histidine–tryptophan–ketoglutarate
INF	initial nonfunction
IRI	ischemia reperfusion injury
KT	kidney transplantation
LT	liver transplantation
mDC	myeloid dendritic cells
Mreg	regulatory macrophages
NK	natural killer
OR	odds ratio
pDC	plasmacytoid dendritic cells
PRA	panel reactive antibodies
ROC	receiver operating characteristics
TAC	Tacrolimus
TCMR	T-cell mediated rejection
Treg	regulatory T cells
WIT	warm ischemia time

## References

[1] Nakamura K., Kageyama S., Kupiec-Weglinski J.W. (2019). Innate immunity in ischemia-reperfusion injury and graft rejection. Curr. Opin. Organ Transpl..

[2] Kezić A., Stajic N., Thaiss F. (2017). Innate Immune Response in Kidney Ischemia/Reperfusion Injury: Potential Target for Therapy. J. Immunol. Res..

[3] Menke J., Sollinger D., Schamberger B., Heemann U., Lutz J. (2014). The effect of ischemia/reperfusion on the kidney graft. Curr. Opin. Organ Transpl..

[4] Zhao D., Abou-Daya K.I., Dai H., Oberbarnscheidt M.H., Li X.C., Lakkis F.G. (2020). Innate Allorecognition and Memory in Transplantation. Front. Immunol..

[5] Béland S., Désy O., Vallin P., Basoni C., De Serres S.A. (2015). Innate immunity in solid organ transplantation: An update and therapeutic opportunities. Expert Rev. Clin. Immunol..

[6] Jo S.-K., Sung S.-A., Cho W.-Y., Go K.-J., Kim H.-K. (2006). Macrophages contribute to the initiation of ischaemic acute renal failure in rats. Nephrol. Dial. Transpl..

[7] Bonventre J.V., Zuk A. (2004). Ischemic acute renal failure: An inflammatory disease?. Kidney Int..

[8] Sepe V., Libetta C., Gregorini M., Rampino T. (2022). The innate immune system in human kidney inflammaging. J. Nephrol..

[9] Zhuang Q., Lakkis F.G. (2015). Dendritic cells and innate immunity in kidney transplantation. Kidney Int..

[10] Lin J., Wang H., Liu C., Cheng A., Deng Q., Zhu H., Chen J. (2021). Dendritic Cells: Versatile Players in Renal Transplantation. Front. Immunol..

[11] Dai H., Thomson A.W., Rogers N.M. (2019). Dendritic Cells as Sensors, Mediators, and Regulators of Ischemic Injury. Front. Immunol..

[12] Rowshani A.T., Vereyken E.J.F. (2012). The Role of Macrophage Lineage Cells in Kidney Graft Rejection and Survival. Transplantation.

[13] Zhai Y., Petrowsky H., Hong J.C., Busuttil R.W., Kupiec-Weglinski J.W. (2013). Ischaemia-reperfusion injury in liver transplantation--from bench to bedside. Nat. Rev. Gastroenterol. Hepatol..

[14] Iovino L., Taddei R., Bindi M.L., Morganti R., Ghinolfi D., Petrini M., Biancofiore G. (2019). Clinical use of an immune monitoring panel in liver transplant recipients: A prospective, observational study. Transpl. Immunol..

[15] Smith S.F., Hosgood S.A., Nicholson M.L. (2019). Ischemia-reperfusion injury in renal transplantation: 3 key signaling pathways in tubular epithelial cells. Kidney Int..

[16] Siedlecki A., Irish W., Brennan D.C. (2011). Delayed graft function in the kidney transplant. Am. J. Transpl..

[17] Solez K., Colvin R.B., Racusen L.C., Haas M., Sis B., Mengel M., Halloran P.F., Baldwin W., Banfi G., Collins A.B. (2008). Banff 07 classification of renal allograft pathology: Updates and future directions. Am. J. Transpl..

[18] Racusen L.C., Solez K., Colvin R.B., Bonsib S.M., Castro M.C., Cavallo T., Croker B.P., Demetris A.J., Drachenberg C.B., Fogo A.B. (1999). The Banff 97 working classification of renal allograft pathology. Kidney Int..

[19] Loupy A., Haas M., Roufosse C., Naesens M., Adam B., Afrouzian M., Akalin E., Alachkar N., Bagnasco S., Becker J.U. (2020). The Banff 2019 Kidney Meeting Report (I): Updates on and clarification of criteria for T cell– and antibody-mediated rejection. Am. J. Transpl..

[20] Boldt A., Borte S., Fricke S., Kentouche K., Emmrich F., Borte M., Kahlenberg F., Sack U. (2014). Eight-color immunophenotyping of T-, B-, and NK-cell subpopulations for characterization of chronic immunodeficiencies. Cytom. Part B Clin. Cytom..

[21] Ladurner R., Steurer W. (2004). Technik der Multiorganentnahme. Viszeralchirurgie.

[22] Eurotransplant Chapter 9: The Donor. https://www.eurotransplant.org/wp-content/uploads/2020/01/H9-The-Donor-Februar-2020.pdf.

[23] Sollinger H.W., Odorico J.S., Knechtle S.J., D’Alessandro A.M., Kalayoglu M., Pirsch J.D. (1998). Experience with 500 simultaneous pancreas-kidney transplants. Ann. Surg..

[24] Sollinger H.W., Odorico J.S., Becker Y.T., D’Alessandro A.M., Pirsch J.D. (2009). One thousand simultaneous pancreas-kidney transplants at a single center with 22-year follow-up. Ann. Surg..

[25] Hosmer D.W., Lemeshow S. (2000). Assessing the Fit of the Model. Applied Logistic Regression.

[26] Leclerc S., Lamarche C. (2021). Cellular therapies in kidney transplantation. Curr. Opin. Nephrol. Hypertens..

[27] Tejchman K., Kotfis K., Sieńko J. (2021). Biomarkers and Mechanisms of Oxidative Stress-Last 20 Years of Research with an Emphasis on Kidney Damage and Renal Transplantation. Int. J. Mol. Sci..

[28] Mócsai A. (2013). Diverse novel functions of neutrophils in immunity, inflammation, and beyond. J. Exp. Med..

[29] Yamamoto S., Nava R.G., Zhu J., Huang H.J., Ibrahim M., Mohanakumar T., Miller M.J., Krupnick A.S., Kreisel D., Gelman A.E. (2012). Cutting edge: Pseudomonas aeruginosa abolishes established lung transplant tolerance by stimulating B7 expression on neutrophils. J. Immunol..

[30] Haug C.E., Colvin R.B., Delmonico F.L., Auchincloss H., Tolkoff-Rubin N., Preffer F.I., Rothlein R., Norris S., Scharschmidt L., Cosimi A.B. (1993). A phase I trial of immunosuppression with anti-ICAM-1 (CD54) mAb in renal allograft recipients. Transplantation.

[31] Salmela K., Wramner L., Ekberg H., Hauser I., Bentdal O., Lins L.E., Isoniemi H., Bäckman L., Persson N., Neumayer H.H. (1999). A randomized multicenter trial of the anti-ICAM-1 monoclonal antibody (enlimomab) for the prevention of acute rejection and delayed onset of graft function in cadaveric renal transplantation: A report of the European Anti-ICAM-1 Renal Transplant Study Gro. Transplantation.

[32] Wang Y., Xu Z., Zhou Y., Xie M., Qi X., Xu Z., Cai Q., Sheng H., Chen E., Zhao B. (2021). Leukocyte cell population data from the blood cell analyzer as a predictive marker for severity of acute pancreatitis. J. Clin. Lab. Anal..

[33] Sekerkova A., Krepsova E., Brabcova E., Slatinska J., Viklicky O., Lanska V., Striz I. (2014). CD14^+^CD16^+^ and CD14^+^CD163^+^ monocyte subpopulations in kidney allograft transplantation. BMC Immunol..

[34] Švachová V., Krupičková L., Novotný M., Fialová M., Mezerová K., Čečrdlová E., Lánská V., Slavčev A., Viklický O., Viklický O. (2021). Changes in phenotypic patterns of blood monocytes after kidney transplantation and during acute rejection. Physiol. Res..

[35] Tinckam K.J., Djurdjev O., Magil A.B. (2005). Glomerular monocytes predict worse outcomes after acute renal allograft rejection independent of C4d status. Kidney Int..

[36] Dooper I.M., Hoitsma A.J., Maass C.N., Assmann K.J., Tax W.J., Koene R.A., Bogman M.J. (1994). The extent of peritubular CD14 staining in renal allografts as an independent immunohistological marker for acute rejection. Transplantation.

[37] Girlanda R., Kleiner D.E., Duan Z., Ford E.A.S., Wright E.C., Mannon R.B., Kirk A.D. (2008). Monocyte infiltration and kidney allograft dysfunction during acute rejection. Am. J. Transpl..

[38] van den Bosch T.P.P., Hilbrands L.B., Kraaijeveld R., Litjens N.H.R., Rezaee F., Nieboer D., Steyerberg E.W., van Gestel J.A., Roelen D.L., Clahsen-van Groningen M.C. (2017). Pretransplant Numbers of CD16^+^ Monocytes as a Novel Biomarker to Predict Acute Rejection After Kidney Transplantation: A Pilot Study. Am. J. Transpl..

[39] Sablik K.A., Litjens N.H.R., Klepper M., Betjes M.G.H. (2019). Increased CD16 expression on NK cells is indicative of antibody-dependent cell-mediated cytotoxicity in chronic-active antibody-mediated rejection. Transpl. Immunol..

[40] Guillén-Gómez E., Guirado L., Belmonte X., Maderuelo A., Santín S., Juarez C., Ars E., Facundo C., Ballarín J.A., Vidal S. (2014). Monocyte implication in renal allograft dysfunction. Clin. Exp. Immunol..

[41] Cho J.-H., Yoon Y.-D., Jang H.M., Kwon E., Jung H.-Y., Choi J.-Y., Park S.-H., Kim Y.-L., Kim H.-K., Huh S. (2015). Immunologic Monitoring of T-Lymphocyte Subsets and Hla-Dr-Positive Monocytes in Kidney Transplant Recipients: A Prospective, Observational Cohort Study. Medicine.

[42] Pontrelli P., Rascio F., Castellano G., Grandaliano G., Gesualdo L., Stallone G. (2020). The Role of Natural Killer Cells in the Immune Response in Kidney Transplantation. Front. Immunol..

[43] Xu X., Han Y., Huang H., Bi L., Kong X., Ma X., Shi B., Xiao L. (2019). Circulating NK cell subsets and NKT-like cells in renal transplant recipients with acute T-cell-mediated renal allograft rejection. Mol. Med. Rep..

[44] López-Botet M., Vilches C., Redondo-Pachón D., Muntasell A., Pupuleku A., Yélamos J., Pascual J., Crespo M. (2017). Dual Role of Natural Killer Cells on Graft Rejection and Control of Cytomegalovirus Infection in Renal Transplantation. Front. Immunol..

[45] Jung H.R., Kim M.J., Wee Y.-M., Kim J.Y., Choi M.Y., Choi J.Y., Kwon H., Jung J.H., Cho Y.M., Go H. (2019). CD56^+^CD^57+^ infiltrates as the most predominant subset of intragraft natural killer cells in renal transplant biopsies with antibody-mediated rejection. Sci. Rep..

[46] Dendle C., Mulley W.R., Holdsworth S. (2019). Can immune biomarkers predict infections in solid organ transplant recipients? A review of current evidence. Transpl. Rev..

[47] Dendle C., Gan P.-Y., Polkinghorne K.R., Ngui J., Stuart R.L., Kanellis J., Thursky K., Mulley W.R., Holdsworth S. (2019). Natural killer cell function predicts severe infection in kidney transplant recipients. Am. J. Transpl..

[48] Fernández-Ruiz M., Silva J.T., López-Medrano F., Allende L.M., San Juan R., Cambra F., Justo I., Paz-Artal E., Jiménez C., Aguado J.M. (2016). Post-transplant monitoring of NK cell counts as a simple approach to predict the occurrence of opportunistic infection in liver transplant recipients. Transpl. Infect. Dis..

[49] Fernández-Ruiz M., López-Medrano F., San Juan R., Allende L.M., Paz-Artal E., Aguado J.M. (2016). Low Natural Killer Cell Counts and Onset of Invasive Fungal Disease After Solid Organ Transplantation. J. Infect. Dis..

[50] Podestà M.A., Cucchiari D., Ponticelli C. (2015). The diverging roles of dendritic cells in kidney allotransplantation. Transpl. Rev..

[51] Zhuang J., Hou J. (2021). The Role of Regulatory Myeloid Cell Therapy in Renal Allograft Rejection. Front. Immunol..

[52] Hackstein H., Renner F.C., Bohnert A., Nockher A., Frommer T., Bein G., Weimer R. (2005). Dendritic cell deficiency in the blood of kidney transplant patients on long-term immunosuppression: Results of a prospective matched-cohort study. Am. J. Transpl..

[53] Nikoueinejad H., Amirzargar A., Sarrafnejad A., Einollahi B., Nafar M., Ahmadpour P., Pour-Reze-Gholi F., Sehat O., Lesanpezeshki M. (2014). Dynamic changes of regulatory T cell and dendritic cell subsets in stable kidney transplant patients: A prospective analysis. Iran. J. Kidney Dis..

[54] Hesselink D.A., Vaessen L.M.B., Hop W.C.J., Schoordijk W., Ijzermans J.N.M., Baan C.C., Weimar W. (2005). The effects of renal transplantation on circulating dendritic cells. Clin. Exp. Immunol..

[55] Batal I., Mohan S., De Serres S.A., Vasilescu E.-R., Tsapepas D., Crew R.J., Patel S.S., Serban G., McCune K., Husain S.A. (2018). Analysis of dendritic cells and ischemia-reperfusion changes in postimplantation renal allograft biopsies may serve as predictors of subsequent rejection episodes. Kidney Int..

[56] Ma L., Liu Y., Wu J., Xu X., Liu F., Feng L., Xie Z., Tang Y., Sun W., Guo H. (2014). Changes in dendritic cells and dendritic cell subpopulations in peripheral blood of recipients during acute rejection after kidney transplantation. Chin. Med. J..

[57] Loverre A., Capobianco C., Stallone G., Infante B., Schena A., Ditonno P., Palazzo S., Battaglia M., Crovace A., Castellano G. (2007). Ischemia-reperfusion injury-induced abnormal dendritic cell traffic in the transplanted kidney with delayed graft function. Kidney Int..

[58] Sun Q., Hall E.C., Huang Y., Chen P., Dibadj K., Murawski M., Shraybman R., Van Kirk K., Tang V., Peng R. (2012). Pre-transplant myeloid dendritic cell deficiency associated with cytomegalovirus infection and death after kidney transplantation. Transpl. Infect. Dis..

[59] Thomson A.W., Ezzelarab M.B. (2020). Generation and functional assessment of nonhuman primate regulatory dendritic cells and their therapeutic efficacy in renal transplantation. Cell. Immunol..

[60] Thomson A.W., Metes D.M., Ezzelarab M.B., Raïch-Regué D. (2019). Regulatory dendritic cells for human organ transplantation. Transpl. Rev..

[61] Sawitzki B., Harden P.N., Reinke P., Moreau A., Hutchinson J.A., Game D.S., Tang Q., Guinan E.C., Battaglia M., Burlingham W.J. (2020). Regulatory cell therapy in kidney transplantation (The ONE Study): A harmonised design and analysis of seven non-randomised, single-arm, phase 1/2A trials. Lancet.

[62] Baci D., Bosi A., Parisi L., Buono G., Mortara L., Ambrosio G., Bruno A. (2020). Innate Immunity Effector Cells as Inflammatory Drivers of Cardiac Fibrosis. Int. J. Mol. Sci..

[63] He Z., Ma C., Yu T., Song J., Leng J., Gu X., Li J. (2019). Activation mechanisms and multifaceted effects of mast cells in ischemia reperfusion injury. Exp. Cell Res..

[64] Yang M., Ma Y., Ding J., Li J. (2014). The role of mast cells in ischemia and reperfusion injury. Inflamm. Res..

[65] Yang M., Ma Y., Tao S., Ding J., Rao L., Jiang H., Li J. (2014). Mast cell degranulation promotes ischemia-reperfusion injury in rat liver. J. Surg. Res..

[66] El-Shitany N., El-desoky K. (2015). Cromoglycate, not ketotifen, ameliorated the injured effect of warm ischemia/reperfusion in rat liver: Role of mast cell degranulation, oxidative stress, proinflammatory cytokine, and inducible nitric oxide synthase. Drug Des. Devel. Ther..

[67] Egido J., Sánchez Crespo M., Lahoz C., García M., de la Concha G., García R., Hernando L. (1980). Evidence of sensitized basophils in renal transplanted patients. Transplantation.

[68] Colvin R.B., Dvorak H.F. (1974). Letter: Basophils and mast cells in renal allograft rejection. Lancet.

[69] Dvorak H.F. (1971). Role of the basophilic leukocyte in allograft rejection. J. Immunol..

[70] Kho M.M.L., Bouvy A.P., Cadogan M., Kraaijeveld R., Baan C.C., Weimar W. (2012). The effect of low and ultra-low dosages Thymoglobulin on peripheral T, B and NK cells in kidney transplant recipients. Transpl. Immunol..

[71] Krepsova E., Tycova I., Sekerkova A., Wohlfahrt P., Hruba P., Striz I., Sawitzki B., Viklicky O. (2015). Effect of induction therapy on the expression of molecular markers associated with rejection and tolerance. BMC Nephrol..

[72] Womer K.L., Peng R., Patton P.R., Murawski M.R., Bucci M., Kaleem A., Schold J., Efron P.A., Hemming A.W., Srinivas T. (2005). The effects of renal transplantation on peripheral blood dendritic cells. Clin. Transpl..

