# Supplementary material for: The Role of Innate Immune Cells in the Prediction of Early Renal Allograft Injury Following Kidney Transplantation
Source: J Clin Med. 2022 Oct 18;11(20):6148. doi: 10.3390/jcm11206148 (PMC9605224; doi:10.3390/jcm11206148)
Supplement: Supplementary file 1 [file jcm-11-06148-s001.zip › jcm-1910308-supplementary.pdf]

Supplementary Materials

Table S1. Frequencies type of induction therapy (with/without ATG) and type of kidney transplantation (living/deceased donor kidney transplantation) with regard to the occurrence of the component endpoint itself (i.e. early renal graft injury (eRGI)), and each single component of the combined endpoint: delayed graft function (DGF), rejection, acute tubular necrosis (ATN), and initial non function (INF).

Variables	Total (N = 50 patients)	eRGI- (N = 27 patients)	eRGI+ (N = 23 patients)	DGF- (N = 33 patients)	DGFI+ (N = 17 patients)	Rejection- (N = 41 patients)	Rejection+ (N = 9 patients)
DD + ATG	4 (8)	1 (4)	3 (13)	2 (4)	2(12)	1 (2.5)	3 (33)
DD - ATG	31 (62)	14 (52)	17 (74)	18 (54)	13 (78)	25 (61)	6 (64)
LD + ATG	1 (2)	1 (4)	0 (0)	1 (3)	0 (0)	1 (2.5)	0 (0)
LD - ATG	14 (28)	11 (40)	3 (13)	12 (39)	2 (12)	13 (34)	0 (0)

Variables	ATN - (n = 35 patients)	ATN + (n = 15 patients)	INF – (n = 50)	INF + (n = 0)
DD+ ATG	1 (3)	3 (26)	4 (8)	0
DD- ATG	20 (57)	11 (67)	31 (62)	0
LD+ ATG	1 (3)	0 (0)	1 (2)	0
LD- ATG	13 (27)	1 (7)	14 (28)	0

Table S2. Uni- and multivariate logistic regression analysis of clinical and immunological predictors for acute (treated) rejection after kidney transplantation.

Variables	UVA	MVA	
	p-Value	OR (95%CI)	*p*-Value
**Recipient factors**			
Recipient age, years	0.01	1.11(1.02-1.21)	0.018
Recipient BMI, <25 versus >25	0.03	NS	NS
Recipient cardiovascular disease (yes versus no)	<0.01	12.3 (1.9-82.3)	<0.01
**Donor factors**			
Donor gender (female versus male)	0.08		
Donor age, years	0.02	1.009 (1.007-1.012)	0.03
donor cerebrovascular disease as cause of death (yes versus no);	0.09		
Donor type- DD und LD	0.03	NS	NS
**Transplant-related factors**			
Cold ischemia time	0.05		
HLA- mismatches >3 versus <3	0.01	1.87 (1.15-3.91)	0.01
Delayed graft function	< 0.01	2.49 (1.57-3.93)	0.03
Number of transplant (re versus first)	0.017	NS	NS
Viral infections (yes versus no)	0.04	NS	NS
bacterial infections	0.03	8.5 (1.5-46.7)	0.019
Average tacrolimus levels, < versus > 8 ng/ml	0.04	NS	NS
**Immunological parameters**			
Neutrophils POD 1; > 12.5 x 10^3/ul	0.025	NS	
Neutrophils POD 7; > 9.4 x10^3/ul	<0.01	18.6 (1.49-216.2)	0.019
Monocytes POD 3; > 850 cell/ul	0.03	NS	
Monocytes POD 7; > 1150 cells/ul	0.023	14.5 (1.14-187.61)	0.039
NK cells POD 3; < 125 cells/ul	<0.01	NS	
NK cells POD 7; < 91 cells/ul	0.018	10.60 (1.11-110.8)	0.031

Basophils POD 3; < 6.7 cells/ul	0.023	9.43 (4.87-45.8)	0.049
Basophils POD 10; < 18.1 cells/ul	0.04	NS	NS
mDC POD 7; < 4.7 cells/ul	<0.01	22.7 (2.61-198.2)	<0.01

Following variables were tested in univariate analysis but failed to show significancy: Recipient gender, donor body mass index; operating time, warm ischemia time, induction therapy (yes versus no), donor (hypertension, diabetes), dialysis modality (hemodialysis versus peritoneal), pre-emptive transplantation (no versus yes); comorbidities recipient (hypertension, diabetes mellitus; peripheral arterial disease); fungal infections, time on dialysis pretransplant; immunological factors: neutrophils POD 0,3 and 10, monocytes POD 0,1 and 10; NK cells on POD 0, 1 and 10; basophils on POD 0,1, and 7; mDC on POD 0,1,3 and 10; pDC on POD 0,1,3,7 and 10; **Abbreviations:** UVA, univariate analysis; MVA, multivariate analysis; OR, odds ratio; CI, confidence interval; BMI, body mass index; NS, not significant; LD, living donor; DD, deceased donor; mDC, myeloid dendritic cells; pDC, plasmocytoid dendritic cells; POD, postoperative days;.

Table S3. Uni- and multivariate logistic regression analysis of clinical and immunological predictors for delayed graft function following kidney transplantation.

**Variables**	**UVA**	**MVA**	
	***p*-Value**	**OR (95%CI)**	***p*-Value**
**Recipient factors**			
Recipient age, years	0.01	1.45 (1.15-1.83)	<0.01
Recipient gender (male versus female)	0.06		
Recipient BMI, <25 versus >25	0.019	1.33 (1.18–1.49)	<0.01
Recipient cardiovascular disease (yes versus no)	0.03	5.28 (1.15-24.3)	0.029
Preemptive transplantation	0.05	NS	NS
**Donor factors**			
Donor gender (female versus male)	0.01	5.9 (1.4-25.6)	0.01
Donor age, years	<0.01	1.13 (1.04-1.23)	<0.01
Donor type- DD versus LD	<0.01	4.1 (1.09-14.8)	0.01
**Transplant-related factors**			
Cold ischemia time	<0.01	1.006(1.001-1.023)	0.02
HLA- mismatches > 3 versus <3	0.07		
Acute rejection	0.02	NS	NS
Average tacrolimus levels, > versus < 8 ng/ml	0.06		
Bacterial infections	0.05	NS	NS
Time on dialyses pretransplant	0.03	NS	NS
Warm ischemia time	0.09		
**Immunological parameters**			
Neutrophils POD 1; > 12.5 x 10^3/ul	0.024	NS	
Neutrophils POD 7; > 9.4 x10^3/ul	0.01	14.0 (1.54-127.2)	0.013
Monocytes POD 1; >725 cells/ul	0.021	NS	
Monocytes POD 3; >850 cells/ul	0.04	NS	
Monocytes POD 7; > 1150 cells/ul	<0.01	23.2 (2.5-311.24)	<0.01
NK cells POD 1; <177 cells/ul	<0.01	NS	
NK cells POD 3; < 125 cells/ul	0.01	5.8 (1.57-29.8)	0.019
NK cells POD 7; < 91 cells/ul	<0.01	NS	
Basophils POD 3; > 6.7 cells/ul	0.045	NS	
Basophils POD 10; < 18.1 cells/ul	0.026	5.21 (1.4–18.9)	0.021
pDC POD 7; < 0.6 cells/ul	0.035	19.8 (1.9-216.2)	<0.01
pDC POD10; <1.0 cells/ul	<0.01	NS	
mDC POD 7; < 4.7 cells/ul	0.03	10.5 (1.01-108.5)	0.018

Following variables were tested in univariate analysis but failed to show significancy: donor body mass index; operating time; induction therapy (yes versus no), donor (hypertension, diabetes), dialysis modality (hemodialysis versus peritoneal), donor cerebrovascular disease as cause of death (yes versus no); comorbidities recipient (hypertension, diabetes mellitus; peripheral arterial disease); fungal infections; viral infections; wating time pretransplant; number of transplant (re versus first); immunological factors: neutrophils POD 0,3 and 10, monocytes POD 0 and 10; NK cells on POD 0 ND 10; basophils on POD 0,1 and 7; pDC on POD 0,1, and 3, mDC on POD 0,1,3 and 10. **Abbreviations:** UVA, univariate analysis; MVA, multivariate analysis; OR, odds ratio; CI, confidence interval; BMI, body mass index; NS, not significant; LD, living donor; DD, deceased donor; mDC, myeloid dendritic cells; pDC, plasmocytoid dendritic cells; POD, postoperative days;.
